# A New Method of Filling Teeth

**Published:** 1884-01

**Authors:** C. A. Timme

**Affiliations:** Hoboken, N. J.


### A NEW METHOD OF FILLING TEETH.

BY DR. C. A. TIMME, HOBOKEN, N. J.

READ BEFORE THE CENTRAL DENTAL ASSOCIATION OF NORTHERN NEW JERSEY.

At the last meeting of the New Jersey State Dental Society, held at Asbury Park, I was commissioned a delegate to the meeting of the Society of German Dentists, to be held at Frankfort-on-the-Main. On arriving at the place of meeting I found that our professional brethren abroad had not been idle, but had made steady advances in practice. Among the new and interesting things that I saw, was a method of filling teeth, in which the so-called magnetic gold is employed.

The originator of the process is Dr. Wilhelm Herbst, of Bremen. Magnetic gold is a form of very soft, and at the same time cohesive gold. Its principal point is its purity. If two pieces of No. 30 or 60 be placed in contact, slightly annealed and then rubbed with a burnisher, it will be found that they have become thoroughly united. It is this high grade of cohesion that has given to it the name of magnetic gold.

In inserting it the cavity is prepared in the usual way, but no retaining points are made, a slight undercut being all that is required. No mallet is used, and the first instruments employed are not unlike the burnishers used for finishing fillings with the dental engine. A few cylinders are placed on the floor of the cavity and against the side walls, the proper point is placed in the engine, and while being rotated it is pressed upon the gold, which is thus easily *burnished* into place. The point must of course be quite clean, and care must be exercised that the friction is not so great as to heat the tooth to a point of discomfort.

When the foundation of the filling is laid another instrument is employed, which may be made by breaking off the head of an engine burr, placing the rest in the engine and grinding the end upon an oiled Arkansas stone. The point not being polished, is, when in use, soon coated with a film of gold. Instead of burnishing the surface therefore, and giving to it a high polish, it merely condenses it and leaves upon it a dead surface that does not prevent the cohesion of fresh pieces. Very little force is employed in the operation, and the point is pressed upon the surface only at interrupted intervals. The engine can therefore be run at a high speed without danger of overheating the tooth.

In this manner gold may be introduced very rapidly, and a large filling may be made in a comparatively short time. The whole is finally finished with heave foil, or with cylinders, and polished in the usual way. The German dentists who are employing this method claim that it is a great improvement upon the old way of filling teeth.

Serrated points not only weaken the gold but they leave a rough uneven surface that it is difficult to make smooth. It is also no doubt true that many soft, weak, poorly organized teeth, can be successfully filled with this gold, that would not withstand the malleting necessary in the use of the usual forms of gold foil ; teeth that were formerly condemned to the use of plastic fillings.

To make a thoroughly good filling with this material considerable practice is necessary, but the method once acquired future operations are greatly facilitated.

Some years since a number of dentists besides myself, attempted to substitute smooth points for serrated ones in the filling of teeth. Our efforts were not, however, crowned with success. We have since discovered that the fault was in the gold, and not with us. Theoretically, smooth points should make a more solid filling, and the gold should be inserted in one-half the time required for the use of serrated points. By the employment of this gold, this theory is proved to be correct in practice, and in Germany it is now extensively employed.

